# Nation, Face, and Identity: An Initial Investigation of National Face in East Asia

**DOI:** 10.3389/fpsyg.2016.01557

**Published:** 2016-10-07

**Authors:** Rong Chen, Kwang-Kuo Hwang

**Affiliations:** Department of Psychology, National Taiwan UniversityTaipei, Taiwan

**Keywords:** face concern, face process, inhibitory effect, national face, national identity, personal face

## Abstract

This research investigates a key concept in East Asia, *face*, and represents the first attempt to empirically examine the concept of face at the national level. Controlling for the level of national identification, Study 1 employed the scenario experiment method among samples of native Chinese and Taiwanese populations and revealed that national face exhibits patterns reverse of personal face. Using the experimental method, Study 2 replicated the findings of Study 1 and provided support for the different mechanisms underneath national face and personal face. Study 3 replicated the findings of Study 2 and additionally showed that national face exerts a significant inhibitory effect on face process. Findings are discussed in terms of possible implications for intergroup and international relations. Expanding on extant scholarship on face and across three studies with different experimental paradigms, this research turns our attention from face at the personal level to face at the national level by introducing the construct of national face and examining its manifestations in East Asia. The results advance our understanding of the psychological mechanism driving face concern in East Asia. They make a strong and unique case for the psychological existence of national face as an empirically distinct construct and an important psychological resource for East Asians.

## Introduction

While the U.S. decided not to join the Chinese-led Asian Infrastructure Investment Bank (AIIB), the reluctance was perceived by Chinese as American response to a threat to face, as the United States gradually loses its predominance in the Asia Pacific (see AIIB, 2015). In her opinion piece for the Council on Foreign Relations, Economy (2015) further stated that “Joining now will be hard to accomplish in a face-saving manner, but the United States could begin by publicly recognizing the need for the financing capabilities in Asia that the AIIB can provide ….” The pivotal role of face in social interactions, particularly in East Asia, has been well noted (e.g., Hwang, 1987), but is there only one type of face? How can the concept of face be applied to the national level? How does face manifest at the national level? What are the role and dynamics of national face? Those are the central questions this paper seeks to address. As such, this research represents the first attempt to empirically examine the concept of face at the national level.

### Face in East Asia

What is *face*? Face is a concept that is intuitively meaningful to people, but one that appears difficult to define precisely. It is concerned with people’s sense of worth, dignity and identity, and is associated with issues such as image, respect, honor, status, reputation and competence (Ting-Toomey and Kurogi, 1998). Moreover, it has simultaneous affective (e.g., feelings of shame and pride), behavioral (e.g., facework), and cognitive (e.g., calculating whether and how much face to give or receive) dimensions (Oetzel et al., 2008). As Brown and Levinson (1987) contended, face is a universal human need.

Although face may not be a concept unique to Asian cultures, scholars have consistently pointed out that concern for face is of utmost importance in most Asian cultures (Hu, 1944; Ho, 1976; Bond, 1993). Indeed, face is originally a concept developed in the Chinese Confucian society as “the most delicate standard by which Chinese social intercourse is regulated” (Lin, 1935, p.200).

According to Hwang (1987, 2012), face plays a key and irreplaceable role in social interactions in Chinese culture. More specifically, face can be differentiated into moral face and social face (Hwang, 2006, 2012). Or, put another way, there are two main sources of face: morality, which is based on one’s moral character, and performance, which entails a person’s social status achieved through successful attainment of life’s goals (Hu, 1944; Cheng, 1986; Ho, 1994).

Furthermore, previous research has shown that, compared with concern for face gain (FG), people attach greater importance to face loss (FL; Zane and Yeh, 2002; Kam and Bond, 2008; Hui and Bond, 2009). This is perhaps, implicitly, people have face as accorded with their roles—unless they lose it. A person can gain face, and one person can *give face* to another, but the major focus is primarily on not losing face (Hamamura et al., 2009; Lin and Yamaguchi, 2011).

### Face at the National Level

Thus far, we have been delineating face at the personal level. While previous research almost exclusively focuses on personal face, however, is there only one type of face? Can we apply the concept to the national level? In April 2001, a Chinese F-8 fighter jet and a U.S. EP-3 spy plane collided over the Hainan Island, for which Beijing blamed the U.S. and expected Washington to apologize and take full responsibility. Both Chinese and Americans viewed the event as a threat to their face (Gries, 2004; Hwang, 2013). After some public outrage and much political maneuvering, the American government increased their regret from *sorry* to *very sorry*, which seemed to be a face-saving way out of the crisis for both sides (Beyond, 2001).

The aforementioned incident presents a fascinating topic for research. In this article we propose that an empirically distinct construct operating at the psychological level, namely *national face*, may help explain such political phenomena. We believe that face exists at the national level, just as it exists at the personal level. As such, it warrants a more direct measure and examination. To the best of our knowledge, no previous study has examined this intriguing topic empirically.

### National Face vs. National Identity

In line with earlier definition, we conceptualize national face as the national self presented to other nations (Gries, 1999). We propose that it entails the feelings of FG/loss that are experienced on the basis of one’s national membership. How is it distinguished from national identity? On a general level, national identity describes the basically positive, subjectively important emotional bond with a nation (Tajfel and Turner, 1986). Previous research has shown that people experience various emotions on behalf of their national group membership (e.g., Smeekes, 2015). Tajfel and Turner (1986) proposed that the groups which people belong to are an important source of pride and self-esteem. In particular, a need for positive distinctiveness drives social identity. Thus, it seems reasonable to expect that the degree to which individuals define themselves in national terms would be closely linked to the concept of national face. However, national face and national identity differ in one crucial respect: public vs. private orientation.

The subjective meaning of identities entails that what it means to be a member of a national group differs for every individual, and it is a private matter in essence (Huddy, 2001). On the other hand, as face must be claimed from other people, there is something essentially public about the conception of face (Kim et al., 2010). As Lim (1994, p. 210) pointed out, “…face is not what one thinks of oneself, but what one thinks others should think of one’s worth.” Notwithstanding, we recognized that national face represents a way in which national identity may be expressed, so in the context of this research, national identity is being treated as a covariant.

As the first attempt to empirically investigate face at the national level, we thought to initially establish and validate the construct of national face through a pilot study.

### Pilot Study: Construct of National Face

The goal of the pilot study was to establish the construct of national face. Drawing on previous research, we envisioned national face to stem from two sources, morality and national performance. We thought to provide initial evidence of the construct of national face and its sources through a pilot study conducted in Taiwan and China. A total of 60 participants, 30 in each country (50% male, *M*_age_ range = 23–30 for both samples), were first recruited from the Taiwanese and mainland Chinese student populations at National Taiwan University, then through the technique of snowball sampling. Abiding by the rule of thumb in the literature (Johanson and Brooks, 2009), we felt the sample size was sufficient for a pilot study.

The participants were asked to fill out a questionnaire which consisted of three open-ended questions: (a) What does the term national face remind you of? (b) Can you give an example of FG in the national context? (c) Can you give an example of FL in the national context? Samples of the answers included (to each question): (a) National image, reputation, status, dignity, collective esteem; (b) Good governance, winning the Olympic gold medal, global recognition of political/economic/cultural strength, tourists’ favorable impressions of the country, upholding international values such as humanitarianism or environmentalism; (c) Loss of sovereignty such as territorial concession, gaffe made by national representatives, losing international negotiations or conflicts, negative international media coverage such as the food contamination scandal, poor infrastructure in the country.

The results suggest that, as expected, national face stemmed from morality and national performance; more specifically, we found that national performance entailed the international and intra-national domains. In short, national face stemmed from three unique sources: (a) morality, i.e., universal values such as humanitarianism and environmentalism, we thus termed them “universal morality,” (b) international performance, e.g., outcome of an international competition, negotiation or conflict, and (c) intra-national performance, e.g., development of local infrastructure, governance, domestic law enforcement.

In addition, the results also reveal that a key channel for the manifestation of national face was the international media. That is, national face concern was activated through international media exposure. This is perhaps not surprising since face inherently entails an image issue (Hwang, 2012), given there is something essentially public about the conception of face as indicated earlier. Goffman’s (1959) dramaturgical theory delineated a connection between the kinds of acts that people put on in their daily lives and theatrical performances. In a social interaction, as in a theatrical performance, there is an on-stage area where actors appear before the audience; this is where positive self-concepts and desired impressions are offered. But there is, as well, a back-stage — a hidden, private area where actors can be themselves and drop their societal roles and identities. In this sense, perhaps the international media serves as the *stage* in accordance with Goffman’s analogy.

In sum, the two major sources of personal face as identified by previous literature, performance and morality, seemed to be applicable in the national context. From the pilot study, we can see that national performance can further be divided into intra-national-related and international-related performances. Hence, national face is constituted from three sources: universal morality (FS1), international performance (FS2), and intra-national performance (FS3).

### From Personal Face to National Face

As Hwang (2012) articulated, a person’s moral face serves as the baseline in Chinese society, meaning it should be maintained in all situations. Indeed, compared with social face, moral face has been found to be of greater concern in Chinese culture (Cheng, 1986; King, 1988; Chu, 1991; Zhai, 1995; Su and Hwang, 2003).

At the national level, however, does this pattern still apply? Or do people attach more concern to face originated from performance? Specifically, would national performance in the intra-national domain be of higher face concern than in the international domain? We suspect that this would be the case given the fact that the Chinese political philosophy is said to be dominated by realpolitik thinking (Deng, 1998; Wang, 2014), resulting from its historical legacy. Specifically, the West’s historic victimization of China, coined the century of humiliation, still looms large in most people’s minds and shapes their worldviews (Cheng and Ngok, 2004). Many believe the international system is characterized by anarchy and power politics. Simply put, a nation has only itself to depend on and thus must ensure its own survival by securing its needs and interests before it looks to the needs of others (Deng, 1998). This implies that in the arena of international politics, where the realities of power and national interest triumph, one realizes that a nation must strengthen domestically before it can be competitive internationally. Hence, we hypothesize face concern for intra-national performance will be significantly higher than face concern for international performance and universal morality, controlling for the level of national identification (Hypothesis 1).

On the other hand, why do people attach less concern to personal FG than personal FL? A large amount of psychological research shows that losses loom larger than gains, a phenomenon referred to as loss aversion; that is, individuals weigh losses more heavily than gains (Kahneman and Tversky, 1979). The concept was originally motivated by the study of choice under uncertainty (Kahneman and Tversky, 1979), but it has since been shown to be applicable across a range of real-world contexts (Camerer, 2000). For example, studies have suggested that losses in income have a larger effect on well-being than equivalent income gains (Boyce et al., 2013). In the case of face, as a Chinese proverb states, “a man needs face like a tree needs bark,” and losing face is “like a tree being stripped of its bark—a life and death situation” (Gao, 1998, p.48). More important, in face cultures, people are supposed to display humility and not overreach on status claims (lest they learn a painful and humiliating lesson about how much status others are willing to accord them), since there is a built-in humility bias (Kurman and Sriram, 2002; Lalwani et al., 2006). Nonetheless, when expanding the face concept to the national level, would one still feel the need to be humble, as the pursuit of greater good triumphs over personal good?

In one line of research, it was found that while one’s acquaintances may share FG resulting from positive events, they do not seem to share FL resulting from negative events. In a study conducted on Taiwanese college students, Liu (2002, unpublished) showed that while the feeling of FG may be contagious, we tend to sever our relationships (with the exception of family members) in the case of FL.

In another line of research, Cialdini et al. (1976) showed that we tend to associate ourselves with winners while disassociate from losers. In a series of field studies, Cialdini et al. (1976) found that some sports fans are happy to support group symbols following their team’s success but rescind their identification following the team’s failure. This phenomenon of basking in reflected glory was further tested in subsequent studies demonstrating the tendency to bask in the reflected glory of another’s success while avoiding the shadow of another’s defeat (Cialdini and De Nicholas, 1989), and that cutting off reflected failure can be distinguished as image-protection strategy for the purpose of avoiding a negative evaluation, while to a lesser degree, basking in reflected glory can be identified as an image-enhancement strategy (Snyder et al., 1986).

Moreover, Bornstein (2003) noted that pride in the group is a public good that is available to all members of a group, in an analysis of the prototypical problems of cooperation and competition within and between groups. Bornstein also noted that group members may have an incentive to *free ride* on the contributions of others. We thus hypothesize that contrary to face concern at the personal level, people will be more concerned with FG than FL at the national level (Hypothesis 2).

### National Face vs. Personal Face

In sum, we expect national face concern to be higher for intra-national performance and FG, just the opposite of personal face concern. To further verify the different mechanisms underlying national face and personal face, we thought to distinguish the two with an experiment. In what conditions do we concern with national face only? In what conditions does personal face come into play? As aforementioned, Goffman’s (1959) dramaturgical theory viewed social interaction as theatrical performance of which actors on stage are being evaluated by the audience. Correspondingly, if the subject is an audience, concern for FG will be higher than FL, since only national face will be involved; however, if the subject is an actor, concern for FL will be higher than FG, since personal face will come into play (Hypothesis 3).

This still begs the question of how national face interplays with personal face. How does face operate at the personal and national levels? Particularly, how does national face concern impact face process? Shi Ke-fa (1601-1645 A.D.), a renowned general in the late Ming dynasty in imperial China, once famously proclaimed that in the case of an army’s defeat, regardless of how bravely the commander has fought, he deserved no commemoration. In the case of a conflicting result between personal and group performances (e.g., personal success/group failure), how does national face concern affect personal face concern?

In order to probe into the effect of national face, we thus thought to investigate national and personal face concerns in conflict vs. non-conflict conditions. Specifically, in a conflict condition, we expect to see an inhibitory effect; in a non-conflict condition, on the other hand, a facilitatory effect is expected to be seen (Hypothesis 4). In addition, since one will attach more concern to national FG (Hypothesis 2), we will witness a higher concern for personal FG than personal FL under such condition, demonstrating a reverse personal face pattern; under the condition of national FL, however, personal face pattern will not be reversed (Hypothesis 5).

## Overview Of Studies

Our research questions are threefold: (a) What is the nature of national face and how does it manifest in East Asia? (b) Are there different mechanisms underneath national face and personal face? (c) How does national face influence face process? The initial aims of the current research are to examine the manifestation of national face in East Asia, and to uncover the effect of national face on face process.

In all societies, people may experience the feeling of gaining or losing face due to positive or negative social evaluation (Hwang, 2006). Put differently, FL/gain denotes a mismatch between an attribute claimed (or denied, in the case of negatively evaluated traits) and an attribute perceived as being ascribed by others (Spencer-Oatey, 2007). Hence conceptually, in the present research, we followed Ho’s (1976) typology to distinguish two kinds of important changes in the status of one’s face: gaining face and losing face (we termed this the “frame” of face concern).

We tested our hypotheses in three studies. In Study 1, we tested Hypotheses 1 and 2. Specifically, having established the construct of national face in the pilot study, we sought to explore the manifestation of national face concern in East Asia by measuring it empirically. To test Hypothesis 3, we examined the processes of national face and personal face in Study 2. In Study 3, to test Hypotheses 4 and 5, we further investigated the effect of national face on face process.

## Study 1: National Face and National Identity in East Asia

In the current study, we sought to explore how national face manifests in Taiwan and China by measuring empirically concerns for the three sources identified in the pilot study, namely, universal morality (FS1), international performance (FS2), and intra-national performance (FS3), and by taking into account the role of national identity. We hypothesize that face concern for intra-national performance (FS3) will be significantly higher than face concern for international performance (FS2) and universal morality (FS1), controlling for the level of national identification (Hypothesis 1). Moreover, concern for FG will be greater than FL (Hypothesis 2).

### Method

#### Participants

A total of 270 participants in China and 248 in Taiwan took part in this study. Among the Taiwanese sample, 123 participants (51% male) completed the FL version of the survey and 125 participants (60% male) completed the FG version of the survey. Among the Chinese sample, 135 participants were surveyed for each version (53% male for the FL version, 39% male for the FG version). For a snapshot of the sample characteristics, please refer to **Table 1**

**Table 1:** Sample characteristics as a percentage of the sample for Study 1.

	Taiwan		China	
Characteristic	FL (*n* = 123)	FG (*n* = 125)	FL (*n* = 135)	FG (*n* = 135)
Gender				
Male	51%	60%	53%	39%
Female	49%	40%	47%	61%
Age					
18–22	22%	15%	7%	5%
23–29	35%	17%	33%	37%
30–39	20%	22%	41%	44%
40–49	8%	17%	18%	13%
50–59	8%	19%	1%	1%
60–65	7%	10%	–	–
Occupation				
Student	50%	21%	7%	6%
Business	8%	19%	35%	32%
Service	15%	17%	18%	20%
IT	6%	10%	3%	5%
Education/research	3%	7%	14%	14%
Government	6%	6%	9%	8%
Others	12%	20%	14%	15%

*FL, face loss; FG, face gain.*

#### Experimental Design

We implemented a 3 × 2 mixed experimental design. The independent measures included face source (FS1 vs. FS2 vs. FS3) as a within-subjects variable and frame (FL vs. FG) as a between-subjects variable.

#### Materials and Procedure

##### National Face Concern

The survey consisted of three scenarios, representing a source of national face apiece conditioned under international media exposure. The scenarios were constructed based on the responses collected in the pilot study. Each scenario was designed to include both FL and FG conditions to make up for the two versions of the survey. For example, the FS1 scenario read (underlined portions represent different wordings for the FL/FG version), “Country C was shattered by an earthquake of unprecedented magnitude, resulting in 100s of refugees awaiting international rescue. Country A neighbors Country C and has close economic ties with Country C. However/Therefore, after the earthquake, Country A neither/immediately joined international rescue work nor/and assisted with any/tons of needed relief supplies; it only/also donated USD$100,000/USD$1 million. Country A’s reactions elicited international media coverage and commentary.”

Participants were randomly assigned to the FL or FG condition. After reading each scenario, participants were asked one manipulation check question presented as check of whether they read and understood the scenario. Each scenario was then followed by three questions relating to the specific events described in order to assess the degree of face concern on a 6-point Likert scale (1 = *strongly disagree*, 6 = *strongly agree*). For example, a question following the above scenario read, “Regarding Country A’s reluctance to come to Country C’s rescue, if Country A was my country, I would feel a loss/gain of national face.”

##### Measure of national identification

In order to assess the participants’ level of national identification, we adopted the national identity scale by Huang (2007), which is an adaptation of the collective self-esteem scale (Luhtanen and Crocker, 1992). Respondents indicated their agreement with statements on a 6-point scale ranging from *strongly disagree* (1) to *strongly agree* (6). Sample items were: “It is very important to me to be able to tell others that I am Taiwanese (Chinese)”; “I will never forget that I am Taiwanese (Chinese)”; “Overall, I enjoy being Taiwanese (Chinese)”; “When others criticize Taiwanese (Chinese), I feel like they are criticizing me.” A total of eight items were included in the scale.

Since samples representative of the population were desired, the surveys were distributed online. Specifically, in Taiwan, we used Survey Monkey and posted links on college bulletin boards; in China, a paid-service was employed to collect data. After seeing a greeting message on the screen, the respondents were instructed to complete the section on national face concern first. After they have finished all the questions, they would then proceed to the section on national identification. Lastly, they were required to answer some demographic questions before leaving the webpage.

### Results and Discussion

We hypothesize that face concern for intra-national performance will be significantly higher than international performance and universal morality, controlling for the level of national identification; moreover, concern for FG will be greater than FL. To test these hypotheses, we performed a Face Source × Frame two-way analysis of covariance (ANCOVA) on the degree of face concern with national identity as covariate using SPSS 20. Higher scores indicated higher concerns for national face. It should be noted that we pooled the data from Taiwan and China in the analyses below because participant characteristics (e.g., age) did not appear to yield any significant differences between the two sample sets.

After adjustment by covariate, the main effect of face source was significant, *F*(2,1030) = 3.88, *MSE* = 0.68, *p* < 0.05, $\eta_{p}^{2}$ = 0.01. The main effect of frame was also significant, *F*(1,515) = 69.13, *MSE* = 1.35, *p* < 0.001, $\eta_{p}^{2}$ = 0.12. Furthermore, the analysis yielded a significant two-way interaction, *F*(2,1030) = 17.31, *MSE* = 0.68, *p* < 0.001, $\eta_{p}^{2}$ = 0.03. The covariate was significantly associated with the dependent variable, *F*(1,515) = 20.75, *MSE* = 1.35, *p* < 0.001, $\eta_{p}^{2}$ = 0.04.

A further analysis showed that the simple main effect of frame was significant under FS1, *F*(1,515) = 71.43, *MSE* = 1.22, *p* < 0.001, $\eta_{p}^{2}$ = 0.12, FS2, *F*(1,515) = 7.40, *MSE* = 0.93, *p* < 0.01, $\eta_{p}^{2}$ = 0.01, and FS3, *F*(1,515) = 40.08, *MSE* = 0.56, *p* < 0.001, $\eta_{p}^{2}$ = 0.07. This demonstrates that across all three sources of national face, concerns for FG were persistently higher than FL. The simple main effect of face source was not significant.

In short, as **Table 2** shows, holding constant the level of national identification (NI = 4.88), face concern for intra-national performance (*M*_FL_ = 4.90, *SD* = 0.05; *M*_FG_ = 5.32, *SD* = 0.05) was significantly higher than face concern for universal morality (*M*_FL_ = 4.06, *SD* = 0.07; *M*_FG_ = 4.88, *SD* = 0.07) and international performance (*M*_FL_ = 4.29, *SD* = 0.06; *M*_FG_ = 4.52, *SD* = 0.06). Moreover, concerns for FG were significantly higher than FL across all three face sources.

**Table 2:** Adjusted marginal means (and SDs) for face source and frame (Study 1).

Variable	FS1	FS2	FS3
FL	4.06^a^ (0.07)	4.29^a^ (0.06)	4.90^a^ (0.05)
FG	4.88^a^ (0.07)	4.52^a^ (0.06)	5.32^a^ (0.05)

*FS1, universal morality; FS2, international performance; FS3, intra-national performance; FL, face loss; FG, face gain. ^a^Covariate (national identity, NI)appearing in the model is evaluated at the following value: NI = 4.88.*

Personal face theory contends that people have greater concern for FL than FG, in addition to valuing moral face more than social face. Notwithstanding, concern for national face yielded just opposite patterns. Are there different mechanisms underneath national face and personal face? In order to further validate the pattern of national face concern and whether there are different psychological mechanisms underneath national face and personal face, we proceeded with an experiment in the next study.

## Study 2: National Face Vs. Personal Face

Having demonstrated the reverse pattern of national face concern for FL and FG in Study 1, in Study 2, we sought to examine whether the process of national face differs from personal face. We propose that if the subject is an actor, then face concern will be higher in the FL condition than in the FG condition; if the subject is an audience, then face concern will be higher in the FG condition than in the FL condition (Hypothesis 3). Since personal face will come into play as the actor himself is in the center of events; by contrast, only national face will be involved as the subject takes no part in the events presented.

### Method

#### Participants

The participants were 32 undergraduate students (47% male, *M*_age_ = 21.24) at National Taiwan University. They took part in the study to receive extra course credit. After giving their consent, the participants received our manipulation instructions and completed a few trials before starting the task. They then answered the manipulation check and demographic questions after task completion.

#### Experimental design

The experimental design was a 2 × 3 within-subjects design. The independent measures included face level (national vs. personal) and frame (FL vs. FG vs. neutral).

#### Materials and Procedure

To distinguish between face concerns at the personal and national levels, we manipulated the roles a participant would play (actor vs. audience). Specifically, participants were first shown a photo of a man, who was described as a national representative, and were asked to play his role. Next, they were informed that they would see a series of photos all involving different national representatives, and to respond accordingly if they themselves appear in the photos; if not, then they were to judge in the role of an audience or citizen. In other words, participants were asked to imagine that they were acting as a state representative and instructed to think about their nation while responding to the items.

The stimuli consisted of 48 photos for each condition. In accordance with the three sources of national face identified in the pilot study, we first selected 16 photos each for the FG and FL conditions, in addition to eight photos for the neutral condition. Next, all photos were digitally altered to include two versions: one of the original and the other of the (manipulated) man. We then used block randomization to compose stimuli for the six conditions in each group; the order of groups was counterbalanced.

In sum, since theoretically, we would only experience personal FL/gain while we ourselves are involved in the situation, alternation of photos was required in order to have the respondents assume the role of the actor (i.e., the manipulated man). In addition to the original set of photos, therefore, each photo was altered to include the manipulated man. Respondents were expected to experience either FG or loss from looking at a series of photos because the photos exemplified FG/loss events with/without the actor (e.g., the national representative being arrested after committing a crime overseas, the national representative yielding serious concessions in an international negotiation, the national representative winning an Olympic Gold Medal, etc.).

Participants were randomly assigned to one of four groups. They were first asked to complete four trials to check whether they understood the instructions. Then, on the computer screen, they saw one photo at a time and indicated their degree of face concern on a Likert scale ranging from -5 (*extreme face loss*) to +5 (*extreme face gain*), with “0” indicating “*not face relevant*.” In the end, they were required to answer four manipulation check questions before filling out basic demographic data.

### Results and Discussion

We hypothesize that if the subject is an actor, then face concern will be higher in the FL condition than in the FG condition; by contrast, if the subject is an audience, then face concern will be higher in the FG condition than in the FL condition. To test this hypothesis, we performed a Face Level (national vs. national + personal) × Frame (FG vs. FL) two-way analysis of variance (ANOVA) on the degree of face concern using SPSS 20. Scores in the neutral condition were subtracted from both FG and FL conditions as they represented a baseline level of face concern for each participant.

The main effect of face level was significant, *F*(1,31) = 14.42, *MSE* = 0.33, *p* < 0.01, $\eta_{p}^{2}$ = 0.32, while the main effect of frame was not significant, *F*(1,31) = 0.75, *MSE* = 0.41, *p* > 0.05, $\eta_{p}^{2}$ = 0.02. The analysis also yielded a significant two-way interaction, *F*(1,31) = 12.34, *MSE* = 0.94, *p* < 0.01, $\eta_{p}^{2}$ = 0.29. A further analysis of simple main effect showed that frame had a significant effect on national face, *F*(1,62) = 11.51, *MSE* = 0.68, *p* < 0.01, $\eta_{p}^{2}$ = 0.19, with higher concern for FG than FL, as well as personal face, *F*(1,62) = 5.98, *MSE* = 0.68, *p* < 0.05, $\eta_{p}^{2}$ = 0.10, with higher concern for FL than FG; face level, on the other hand, had a significant effect only under the FL condition, *F*(1,62) = 24.42, *MSE* = 0.64, *p* < 0.001, $\eta_{p}^{2}$ = 0.40, with concern for personal FL higher than national FL.

In short, consistent with the results in Study 1, concern was higher for FG than FL at the national level. We also provided further support for the argument that at the personal level, concern was higher for FL than FG (see **Table 3** below). These findings validate that there are different processes for national face and personal face, while they interact with each other.

**Table 3:** Means (and SDs) for face level and frame (Study 2).

Variable	NF	NF + PF	Neutral
FL	2.10 (0.93)	3.09 (0.75)	
FG	2.80 (0.86)	2.58 (0.93)	
Neutral			0.51 (0.54)

*NF, national face; PF, personal face; FL, face loss; FG, face gain.*

Although we were able to replicate the results of Study 1 in the present experiment, we were still unclear of how national face interplays with personal face and the effect of national face on face process. We thus conducted the next study to investigate this issue.

## Study 3: The Effect of National Face

As we have revealed the reverse patterns of national face and personal face in Studies 1 and 2, in Study 3, we extended the results of Experiments 1 and 2 to a slightly different paradigm so that we can examine the crucial role national face plays in face process.

How does national face interact with personal face to influence face process? We hypothesize that in a conflict condition, national face will affect face process through an inhibitory effect; in a non-conflict condition, national face will affect face process through a facilitatory effect (Hypothesis 4). Furthermore, under NFL (national FL), personal face pattern will remain the same, i.e., concern for PFL (personal FL) will be greater than PFG (personal FG); however, under NFG (national FG), concern for PFG will be greater than PFL, thus reversing personal face pattern (Hypothesis 5).

### Method

#### Participants

The participants in Study 3 were 35 undergraduate students at National Taiwan University (45% male, *M*_age_ = 20.30). They received extra course credit for their participation.

#### Experimental Design

The current study involved a 2 × 3 experimental design, including frame of national face (NFG vs. NFL) and frame of personal face (PFG vs. PFL vs. control) as within-subjects variables. Since our main interest lay in the conflict vs. non-conflict condition between face level and frame, we also added a control group with gain/loss of personal face only.

#### Materials and Procedure

The stimuli contained 40 scenarios written in paired- and single sentences, all controlled for sentence length (in Chinese). For example, a scenario for the NFL/PFG condition read, “Tourists from our nation have been rated the most unwelcomed tourists around the world. But I always mind my manners when traveling abroad.” A scenario for the NFG/PFL condition read, “Our nation is regarded as a global sports giant. But the tae kwon do competition for which I represent has never won any major international medals.” A scenario for the NFL/PFL condition read, “Our public transports are known for being late and for frequent violation of traffic rules. As a driver I also often violate traffic rules and run the red light.” A scenario for the NFG/PFG condition read, “Our nation has been a leader in global humanitarian relief. I led the volunteer work when a neighboring country was struck by a major tsunami.” A scenario for the PFL condition read, “I was caught fabricating research data in order to publish in academic journals.” A scenario for the PFG condition read, “I invented a pioneering water recycling technique.”

The order of stimuli was counterbalanced and participants were randomly assigned to one of two groups. After giving their consent, they read one scenario on the computer screen at a time and indicated their degree of face concern on a Likert scale ranging from -5 (*extreme face loss*) to +5 (*extreme face gain*), with “0” indicating “*not face relevant*.” In the end, they were required to answer a few demographic questions.

### Results and Discussion

We hypothesize that in a conflict condition, national face will affect face process through an inhibitory effect; in a non-conflict condition, national face will affect face process through a facilitatory effect. Moreover, concern for PFL will be greater than PFG under NFL; however, concern for PFG will be greater than PFL under NFG, a reverse of personal face pattern. To test these hypotheses, we performed a Frame of National Face (NFG vs. NFL) × Frame of Personal Face (PFG vs. PFL vs. control) two-way analysis of variance (ANOVA) on face concern using SPSS 20. The main effect of frame of national face was significant, *F*(2,68) = 31.37, *MSE* = 0.42, *p* < 0.001, $\eta_{p}^{2}$ = 0.48. The main effect of frame of personal face was also significant, *F*(1,34) = 11.61, *MSE* = 0.64, *p* < 0.01, $\eta_{p}^{2}$ = 0.26. Moreover, the analysis yielded a significant two-way interaction, *F*(2,68) = 66.60, *MSE* = 0.61, *p* < 0.001, $\eta_{p}^{2}$ = 0.66.

A further analysis of simple main effect demonstrated that when the process of PFL was involved, concern for NFL was significantly higher than NFG, *F*(2,136) = 30.83, *MSE* = 0.79, *p* < 0.001, $\eta_{p}^{2}$ = 0.45; on the other hand, when the process of PFG was involved, concern for NFG was significantly higher than NFL, *F*(2,136) = 36.68, *MSE* = 0.79, *p* < 0.001, $\eta_{p}^{2}$ = 0.54.

Does this suggest that national face played a rather minor role in face process? We think not. As **Table 4** shows, under NFL, the degree of concern for PFL was greater than PFG (*M*_PFL_ = -3.01, *SD* = 0.90 vs. *M*_PFG_ = 1.18, *SD* = 0.76); however, under NFG, the degree of concern for PFG was greater than PFL (*M*_PFG_ = 2.83, *SD* = 0.83 vs. *M*_PFL_ = -1.64, *SD* = 1.08), suggesting a reverse of personal face pattern under NFG.

**Table 4:** Means (and SDs) for frame of NF and frame of PF (Study 3).

Variable	NFG	NFL	Control
PFG	2.83 (0.83)	1.18 (0.76)	2.66 (0.92)
PFL	-1.64 (1.08)	-3.01 (0.90)	-3.15 (0.91)

*NFG, national face gain; NFL, national face loss; PFG, personal face gain; PFL, personal face loss.*

Analysis of simple main effect also showed that when the process of NFG was involved, concern for PFG was significantly higher than PFL, *F*(1,102) = 28.68, *MSE* = 0.87, *p* < 0.001, $\eta_{p}^{2}$ = 0.28, a pattern opposite of that suggested by personal face theory (e.g., Hwang, 2012). This demonstrates that the process of national face had a significant impact on face concern.

Moreover, comparing with results of the control condition (which only involved personal face process), in a non-conflict condition (e.g., NFG/PFG), the interaction was not significant; interestingly, in a conflict condition (e.g., NFG/PFL), national face exerted a significant inhibitory effect on personal face (see **Table 5** below). This suggests that under PFL, when NFG was involved, the degree of face concern would lessen, *F*(2,136) = 30.83, *MSE* = 0.79, *p* < 0.001, $\eta_{p}^{2}$ = 0.45; similarly, under PFG, when NFL was involved, the degree of face concern would also lessen, *F*(2,136) = 36.68, *MSE* = 0.79, *p* < 0.001, $\eta_{p}^{2}$ = 0.54. In short, we replicated and extended the results of Studies 1 and 2 showing that, national face had a significant impact on face process through an inhibitory effect.

**Table 5:** Mechanism of national face and personal face in non-conflict vs. conflict conditions.

	NFG	NFL
PFG	None	Inhibitory national face effect
PFL	Inhibitory national face effect	None

*NFG, national face gain; NFL, national face loss; PFG, personal face gain; PFL, personal face loss.*

## Summary and General Discussion

Humans live in a cultural context. Numerous studies have demonstrated differences between East Asia and the West on perception and cognition, for instance, and highlighted the cultural reasons for their results. Indeed, as Norenzayan et al. (2010) pointed out, cultural differences influence the content of minds, or the domains of thinking to which cognitive strategies are applied? And these cultural differences are tied to different construals of the self, ecological differences in visual environments, in assumptions about the nature of the world, in beliefs about the origins of knowledge, in linguistic conventions, in expertise or familiarity with certain domains of life but not others, and in social practices that promote some cognitive strategies at the expense of others.

Although the concept of face is not confined to a specific culture, how people shape the meaning of face differs from one culture to another. Nearly all researchers across the East and West identify face as a major dimension of East Asian culture. East Asians are sensitive to face issues because of the cultural emphasis on enduring relationships and social networks. Due to the influence of Confucianism, which focuses on the morality and ideals of human relationships, the traditional self is viewed as relations with others, and face in East Asian culture stands for the social-self face of a big group (Hwang, 1987). Accordingly, one characteristic of face in East Asia is that it is shared. For instance, Chinese often talk of everyone having face, suggesting that if one member of a group loses face, the entire group loses face (Cardon and Scott, 2003). That is, face often refers to entire groups. Hence, it is possible to speak about the face of the Chinese people. Groups maintain a status or reputation, and individuals are concerned about not only their personal face but also the face of their groups (Jia, 2001). Therefore, East Asians tend to have strong face consciousness.

Indeed, over seven decades ago in a seminal article, Hu (1944, pp. 48, 50, 59) gave various examples of face where the referent object was the nation rather than the individual: (1) The appeasement policy under Neville Chamberlain had led to a loss of British face in the eyes of the Chinese. (2) Chinese locals were concerned about losing “the face of their country” when dealing with Americans. (3) During the Sino-Japanese war, the Chinese saw the British as “padding China’s face.”

In sum, the pivotal role of face in social interactions in East Asia has been well documented (Zhang et al., 2011; Hwang, 2012). Notwithstanding, much of the discussion has centered on face at the personal level. How can the concept be applied to the national level? The current research represents the first attempt to empirically investigate the construct and role of national face. In the present studies, we examined some very simple situations involving national face concern. Yet, even in these simple situations, national face proved to be an empirically distinct construct as it displayed a very different pattern compared with personal face.

The contributions of the current research are thus twofold. First, it extends extant literature by introducing the construct of national face and providing support for the need for national face, beyond national identity, in East Asia. Second, the current research unveils the reverse pattern of national face vis-a-vis personal face and its significant inhibitory effect on face process. This investigation is theoretically significant because it sheds light on the underpinnings of national face and deepens our understanding of the potential consequences of national face on face process. In sum, the results advance our understanding of the psychological mechanism driving face concern in East Asia. They make a strong and unique case for the psychological existence of national face as an important psychological resource for East Asians, particularly in the perception of events in the context of international relations.

### Understanding National Face

From the pilot study, we can see that at the national level, the three unique sources of face are: universal morality, international performance, and intra-national performance. Furthermore, Study 1 demonstrates that in East Asia, face for intra-national performance is of utmost concern. The results also indicate that, just the reverse of personal face pattern, FG at the national level is of greater concern than FL. At the personal level, morality serves as the source for greater face concern; notwithstanding, at the national level, as hypothesized, performance—particularly in the intra-national domain—takes priority in the realities of power politics.

**FIGURE 1:**
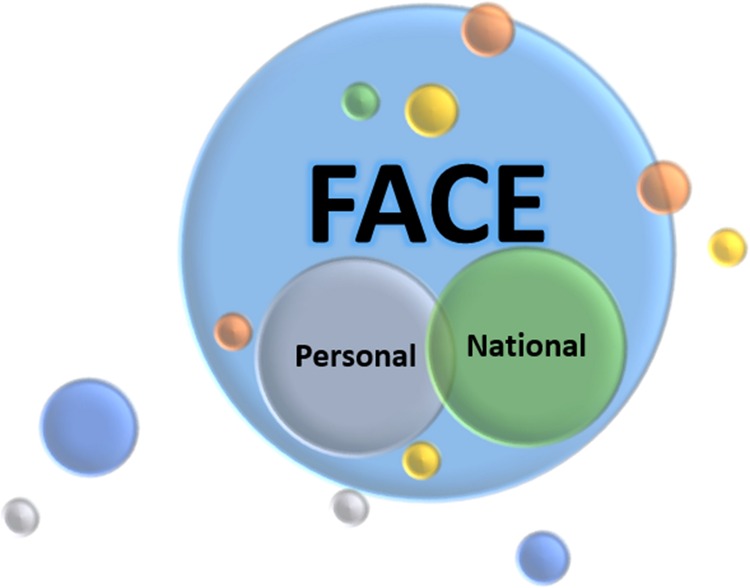
**Two types of face, as represented at the personal and national levels**.

Consistent with the results in Study 1, Studies 2 and 3 show that national face exerts a pattern reverse of personal face, i.e., concern for FG is greater than FL at the national level. In line with our hypotheses, people have the tendency to associate with winners while dissociate from losers. The results also allow us insight into the different psychological mechanisms underlying national face and personal face. In particular, our research shows a significant inhibitory effect of national face on face process. Taken together, we believe the findings make a strong and unique case for the psychological existence of national face as an empirically distinct construct and important psychological resource for East Asians. As **Figure 1** shows, the concept of face exists not only at the personal level, but also at the national level.

### National Face and National Identity

According to social identity theory (Tajfel and Turner, 1986), a need for positive distinctiveness drives social identity. Our research demonstrates that the need for national face serves a significant motive beyond the identification with our national group in East Asia. Importantly, our results show that national face is empirically distinct from the potentially related construct of national identity. We believe this to be an original and critical finding, which yields unique contribution to social identity theories in general and to national identity theories in particular.

### Limitations and Future Research

One limitation of this research is that we have sampled from East Asia. Can our findings be generalized to other cultures? Indeed, the concept of face is not only salient in Asia, but is also of universal nature (Ho, 1976; Ting-Toomey and Kurogi, 1998). As such, replication in other countries is needed to determine whether the findings obtained in this research can be reliably generalized to other cultural contexts.

This research turns our attention from face at the personal level to face at the national level. A critical review of previous literature reveals that the overwhelming focus has been on examining personal face experiences rather than cognition (e.g., Choi et al., 1997). In addition to exploring the nature of national face, the current paper emphasizes the psychological mechanism and intrapersonal processes of face from the perspective of the audience, rather than the actor. Future research could combine investigation of these processes among lay people, as we did in our experiments, with empirical investigations among political elites, whose national face concern might more directly influence political decision making.

What are the implications of national face concern for international relations? How does national face shape the perceptions, attitudes, and behaviors in international relations? What are its consequences? Do concerns over national face motivate nationalistic tendency, as Gries (1999, 2004) suggested in the term Chinese “face nationalism?” Future research could explore the consequences of national FL/gain in an international relations context, in order to promote intergroup understanding and relations.

In a sense, one might find our results to be interestingly counterintuitive, since it appeared that many controversies or conflicts in international relations in recent years were originated when a country felt to have their face threatened. Yet, our findings demonstrate that there is more concern about FG than FL. One explanation could be the fact that FL conditions often entail consequences for which countries feel the need to resort to means of face saving, thereby generating more public attention, whereas no actions would be deemed necessary after a FG on achievements.

It is important to note that, in this paper, one may find the concept of national face to be parallel to national image. The two concepts do share many similar aspects as illustrated in the discussions above; however, a key distinction between them is that face can be exchanged (Hwang, 2012). For example, one may intend to *give face* to someone as a favor; in fact, reciprocity is considered a core element of face and plays a key role in Chinese social interaction (Hwang, 1987). One’s image, on the other hand, cannot be exchanged. How would this element play out at the national level (for instance, in an international negotiation) remains an intriguing topic for future research.

## Conclusion

Living in China for over 20 years, German missionary and scholar Richard Wilhelm (1873–1930), who first pointed out the cultural origin of face, has noted almost a century ago that concern for face is intricate and deeply rooted in Chinese culture (as cited in Hwang, 2012). The current research investigates a key concept in East Asia, face, and represents the first attempt to empirically examine the concept of face at the national level. Expanding on extant scholarship on face and across three studies with different experimental paradigms, this research turns our attention from face at the personal level to face at the national level by introducing the construct of national face and examining its manifestation in East Asia. The results advance our understanding of the psychological mechanism driving face concern. They not only indicate how the concept of face can be extended to the national level, but also make a strong and unique case for the psychological existence of national face as an important psychological resource for East Asians, particularly in the perception of events in the context of international relations. To our knowledge, this is the first empirical evidence of how face can be applied at the national level.

Although the initial focus is on psychological mechanism and intra-personal process, we hope that this first probe into the construct and role of national face will help bridge the gap between the social psychological science on the one hand, and political psychology on the other hand. We hope that insights from the current investigation will contribute new social psychological contents to the literature on face and identity, and inspire future studies in which a key concept that may be connected to international relations is examined. The ultimate goal is to increase cross-cultural understanding in the hope of enhancing intergroup relations, and perhaps even contribute to the reduction of global conflict.

## Author Note

Part of this paper was presented at the 38th Annual Scientific Meeting of the International Society of Political Psychology in San Diego, CA, USA, July 3–6, 2015, and at the 11th Biennial Conference of Asian Association of Social Psychology in Cebu, Philippines, August 19–22, 2015.

## Author Contributions

RC is the first author of this article. K-KH gave the final approval of the version to be published.

## Conflict of Interest Statement

The authors declare that the research was conducted in the absence of any commercial or financial relationships that could be construed as a potential conflict of interest.
